# 

**Published:** 1885-01

**Authors:** 


### CONTENTS.

ORIGINAL COMMUNICATIONS: PAGE.

I. A Case of Mural Pregnancy. By William H. Byford, M. D., Professor of Gynecology, Rush Medical College, Chicago ... 1

II. Psoriasis. A Clinic at the College of Physicians and Surgeons of Chicago. By Henry J. Reynolds, M. D., Professor of Dermatology ... 7

III. Is Piric Acid a Reliable and Practical Test for Albuminuria? By Frank Cary, M. D., Chicago ... 9

IV. A Simple Microtome. By C. C. Webster, M. D., Chicago ... 12

EDITORIAL:

The External Use of Chloroform in Labor ... 14

Physician's Protective Association ... 15

NEWS ITEMS ... 17

BOOK REVIEWS:

A Practical Treatise on Disease in Children By Eustace Smith, M. D., F. R. C. P., Physician to His Majesty the King of the Belgians, Physician to the East London Children's Hospital, and to the Victoria Park Hospital for Diseases of the Chest. Large 8vo., pp. 884. New York: William Wood & Co., Publishers ... 20

FOREIGN CORRESPONDENCE:

University of Aberdeen, Scotland ... 24

Letter from Weisbaden, Germany ... 30

Letter from Italy ... 37

SELECTIONS:

The Will of Sir Erasmus Wilson ... 40

Committee on Organization of the Ninth International Medical Congress, to be held in Washington, D. C., in 1887. Preliminary Notice ... 42

Report of Proceedings of the Illinois State Board of Health, Quarterly Meeting, Nov. 20–21, 1884 ... 46

SOCIETY PROCEEDINGS:

Chicago Gynecological Society ... 58

Chicago Medical Society ... 67

